# New pathogen for gastric cancer: *Streptococcus anginosus*

**DOI:** 10.1002/ctm2.70104

**Published:** 2024-11-28

**Authors:** Ruijie Zeng, Joseph J. Y. Sung, Jun Yu

**Affiliations:** ^1^ Institute of Digestive Disease and Department of Medicine and Therapeutics, State Key Laboratory of Digestive Disease, Li Ka Shing Institute of Health Sciences, CUHK Shenzhen Research Institute The Chinese University of Hong Kong Hong Kong SAR China; ^2^ Lee Kong Chian School of Medicine Nanyang Technological University Singapore

1

It is well‐established that *Helicobacter pylori* is the major risk factor for gastric cancer (GC). For the past decades, overwhelming studies have focused on *H. pylori* to elucidate its procarcinogenic mechanisms and establish eradicating modalities. However, *H. pylori* triggers GC in a “hit and run” manner and largely depletes, rather than persistently involved in gastric tumorigenesis and progression. When gastric intestinal metaplasia occurs, *H. pylori* eradication fails to reduce the risk of GC.^1^ In addition, less than 3% of *H. pylori*‐infected individuals eventually develop GC,^2^ and there should be more contributing factors being taken into account in addition to *H. pylori*. As gastric tumorigenesis initiates, impaired gastric acid and enzyme secretions facilitate the colonization of diverse microbes. The roles and mechanisms of non‐*H. pylori* microbes in GC have long been underrecognized.

To thoroughly depict the non‐*H. pylori* microbiome in GC, we have assessed microbial alterations across various stages of gastric tumorigenesis and identified that *Streptococcus anginosus* is notably associated with atrophic gastritis, intestinal metaplasia, and GC.^3^ After *H. pylori*‐eradication, *S. anginosus* still contributes to persistent gastric inflammation.^4^ Evidence consistently suggests the involvement of *S. anginosus* in gastric tumorigenesis, prompting us to investigate its effects and mechanisms.^5^

Our findings show that *S. anginosus* can colonize within the stomach of mice. *S. anginosus*‐infected mice exhibit acute gastric mucosal inflammation under pro‐inflammatory cytokine production after 2 weeks. Chronic inflammation and precancerous lesions, including atrophy, metaplasia, and low‐grade dysplasia occur under *S. anginosus* long‐term exposure, which is similar to the Correa's cascade observed in patients with GC. *S. anginosus*‐infection of germ‐free mice demonstrates similar results, indicating the pro‐inflammatory and pro‐tumorigenic effects of *S. anginosus* can be sufficient without the involvement of other microbes. In addition, *S. anginosus* accelerates gastric tumorigenesis in both short‐term allograft and long‐term chemical‐induced GC models. Moreover, *S. anginosus* infection induces CD8^+^ T cell exhaustion as well as immunosuppressive cell infiltrations as revealed by single‐cell RNA sequencing. Mechanistically, through screening by pull‐down assays and mass spectrometry, the surface adhesin TMPC from *S. anginosus* binds to the annexin A2 receptor on gastric cells, which further activates the mitogen‐activated protein kinase (MAPK) signalling. The pro‐tumorigenic effects can be abolished through ANXA2 knockout on GC cells or TMPC deletion within *S. anginosus*. These findings are highly relevant to a variety of issues we have encountered clinically (Figure 1).

**FIGURE 1:**
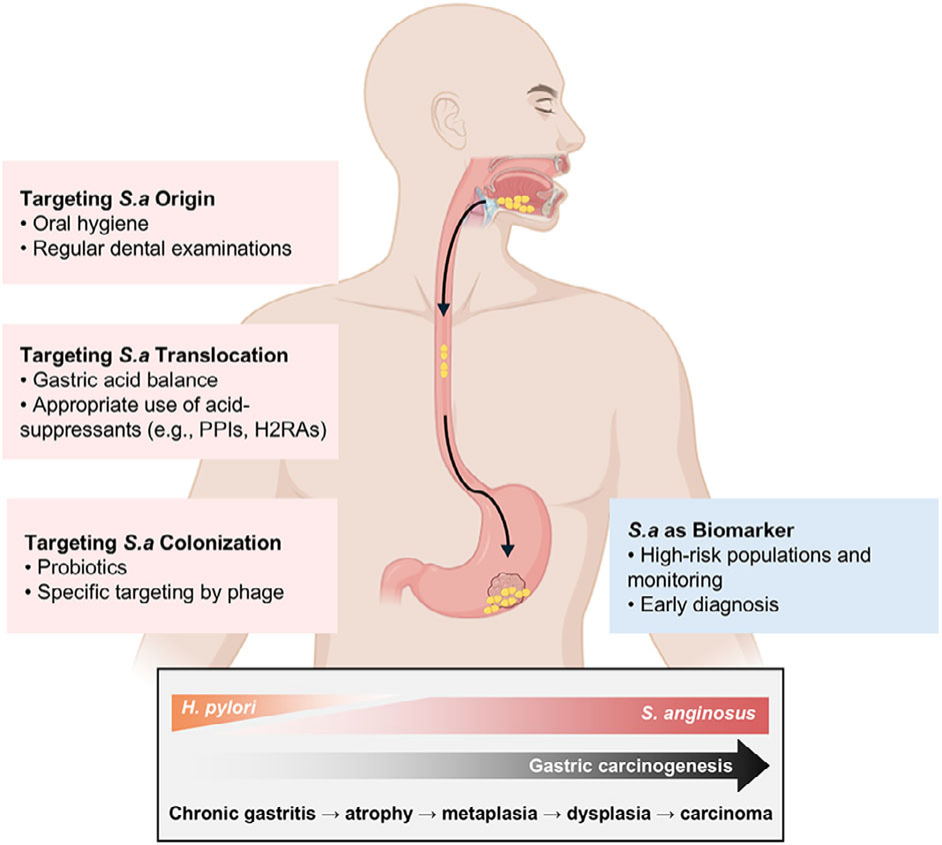
Potential clinical implications related to *Streptococcus anginosus* (*S. anginosus*) and gastric cancer (GC). Compared to *Helicobacter pylori* (*H. pylori*) which mainly serves as a trigger and induces chronic gastritis, *S. anginosus* is involved throughout different stages of gastric tumorigenesis. *S. anginosus* is a promising biomarker for early detection of GC. Potential interventions targeting the origin, translocation, and colonization of *S. anginosus* can be taken to prevent its overabundance and pathogenesis in the stomach. H2RA: histamine type‐2 receptor antagonists; PPI: proton pump inhibitor.

Prevention is always better than cure. In clinical practice, biomarkers for early detection of GC remain scarce, and a large number of patients are diagnosed at their advanced stages with short survival. Detection of gastric microbiota may open a new area for identifying high‐risk populations for GC.

Modifiable risk factors are disease‐related exposures that can potentially be changed, whereas only a limited number of GC‐associated risk factors are modifiable. *S. anginosus* represents a new modifiable risk factor and is promising to be controlled for suppressing gastric tumorigenesis. *S. anginosus* mainly originates from the oral cavity, particularly in patients with oral infections such as gingivitis and dental abscesses.^6^ Therefore, regular dental examinations and maintaining oral hygiene are the simplest and most cost‐effective ways to prevent the overabundance of oral pathogens including *S. anginosus* within the oral cavity.

Gastric acid is bactericidal to pathogens, while impaired acid secretion might facilitate *S. anginosus* translocation and colonization within the stomach. Apart from pathological changes, acid suppressant use is a common factor for decreased acid secretion. It is estimated that approximately 10% of the population in the United States is using acid suppressants, and their inappropriate use without clear indications has raised extensive concerns. Intriguingly, acid‐suppressants induce gut microbial alterations, with *S. anginosus* being the most significantly changed species.^7^ Epidemiological studies also indicate that acid suppressants are related to a higher risk of GC.^8^ Acid‐suppressant use, *S. anginosus* translocation, and subsequent GC occurrence are tightly linked together based on current evidence. Thus, appropriate use and de‐prescribing of acid‐suppressants is essential to ameliorating the burden and mitigating the risks of adverse effects.

Specifically targeting *S. anginosus* in clinical practice remains unfeasible. Antibiotics are non‐specific to *S. anginosus* and will disturb normal gut flora.^9^ In addition, antibiotics do not guarantee the complete clearance of *S. anginosus* within the oral cavity and gastrointestinal tract, and the emergence of drug‐resistant *S. anginosus* or other pathogenic strains will be much more intractable. In contrast, probiotics that antagonize *S. anginosus* represent promising alternatives, and targeted therapy using phage is another viable strategy against *S. anginosus*. Further investigations are warranted into this field to explore their effectiveness and mechanisms.

Taken together, *S. anginosus* is a new promoter and accelerator involved in GC tumorigenesis throughout different stages. Our findings might be merely the tip of the iceberg of non‐*H. pylori* microbes in gastric tumorigenesis, and will inspire researchers and clinicians to delve deeper into the concealed parts of gastric microbiota.

## AUTHOR CONTRIBUTIONS

RZ designed the figures and drafted the manuscript. JY and JJS supervised the study and revised the figures and manuscript. All authors approved the final version of the manuscript.

## CONFLICT OF INTEREST STATEMENT

The authors declare no conflict of interest.

## References

[1] Chen HN , Wang Z , Li X , Zhou ZG . Helicobacter pylori eradication cannot reduce the risk of gastric cancer in patients with intestinal metaplasia and dysplasia: evidence from a meta‐analysis. Gastric Cancer. 2016;19(1):166‐175. doi:10.1007/s10120-015-0462-7 25609452

[2] Kumar S , Metz DC , Ellenberg S , Kaplan DE , Goldberg DS . Risk factors and incidence of gastric cancer after detection of *Helicobacter pylori* infection: a large cohort study. Gastroenterology. 2020;158(3):527‐536.e7. doi:10.1053/j.gastro.2019.10.019 31654635 PMC7010558

[3] Coker OO , Dai Z , Nie Y , et al. Mucosal microbiome dysbiosis in gastric carcinogenesis. Gut. 2018;67(6):1024‐1032. doi:10.1136/gutjnl-2017-314281 28765474 PMC5969346

[4] Sung JJY , Coker OO , Chu E , et al. Gastric microbes associated with gastric inflammation, atrophy and intestinal metaplasia 1 year after *Helicobacter pylori* eradication. Gut. 2020;69(9):1572‐1580. doi:10.1136/gutjnl-2019-319826 31974133 PMC7456733

[5] Fu K , Cheung AHK , Wong CC , et al. Streptococcus anginosus promotes gastric inflammation, atrophy, and tumorigenesis in mice. Cell. 2024;187(4):882‐896.e17. 38295787 10.1016/j.cell.2024.01.004

[6] Terzi HA , Demiray T , Koroglu M , et al. Intra‐abdominal abscess and primary peritonitis caused by *Streptococcus anginosus* . Jundishapur J Microbiol. 2016;9(6):e33863. 27630763 10.5812/jjm.33863 PMC5011413

[7] Xiao X , Zhang X , Wang J , et al. Proton pump inhibitors alter gut microbiota by promoting oral microbiota translocation: a prospective interventional study. Gut. 2024;73(7):1098‐1109. doi:10.1136/gutjnl-2023-330883 38267200

[8] Zeng R , Sha W , Wang J , et al. Evaluation of proton pump inhibitors and risks of gastric cancer. Gut. 2022;71(9):1924‐1926. doi:10.1136/gutjnl-2021-326291 34836917

[9] Knoop KA , McDonald KG , Kulkarni DH , Newberry RD . Antibiotics promote inflammation through the translocation of native commensal colonic bacteria. Gut. 2016;65(7):1100‐1109. 26045138 10.1136/gutjnl-2014-309059 PMC4670297

